# Predicting the Coupling Properties of Axially-Textured Materials

**DOI:** 10.3390/ma6114967

**Published:** 2013-10-30

**Authors:** Luis E. Fuentes-Cobas, Alejandro Muñoz-Romero, María E. Montero-Cabrera, Luis Fuentes-Montero, María E. Fuentes-Montero

**Affiliations:** 1 Centro de Investigación en Materiales Avanzados, Miguel de Cervantes 120, Chihuahua, Chih 31109, Mexico; E-Mail: elena.montero@cimav.edu.mx; 2 Delphi Automotive Systems, The Mexico Technical Center, Av Hermanos Escobar No 5756, Ciudad Juarez, Chih 32310, Mexico; E-Mail: alejandro.munoz07@gmail.com; 3 Diamond Light Source Ltd., Diamond House, Harwell Science and Innovation Campus, Didcot, Oxfordshire OX11 0DE, UK; E-Mail: luis.fuentes-montero@diamond.ac.uk; 4 Facultad de Ciencias Químicas, Universidad Autónoma de Chihuahua, Nuevo Campus Universitario, Circuito Universitario Chihuahua, Chih 31125, Mexico; E-Mail: mfuentes@uach.mx

**Keywords:** properties prediction, polycrystals, texture, piezoelectricity, computer modeling

## Abstract

A description of methods and computer programs for the prediction of “coupling properties” in axially-textured polycrystals is presented. Starting data are the single-crystal properties, texture and stereography. The validity and proper protocols for applying the Voigt, Reuss and Hill approximations to estimate coupling properties effective values is analyzed. Working algorithms for predicting mentioned averages are given. Bunge’s symmetrized spherical harmonics expansion of orientation distribution functions, inverse pole figures and (single and polycrystals) physical properties is applied in all stages of the proposed methodology. The established mathematical route has been systematized in a working computer program. The discussion of piezoelectricity in a representative textured ferro-piezoelectric ceramic illustrates the application of the proposed methodology. Polycrystal coupling properties, predicted by the suggested route, are fairly close to experimentally measured ones.

## 1. Introduction

Crystallographic texture plays a significant role on the physical properties of bulk and nano-structured materials. Predicting the influence of texture on the materials’ properties is a powerful tool in engineering design [1,2]. The quantitative characterization of the mentioned effect has been focused, significantly, on the prediction of the mechanical properties of metals and alloys [3,4,5]. One of the most important areas of opportunity in materials science, nowadays, is the field of functional materials. Materials that convert one type of stimulus (mechanical, thermal, magnetic, luminous, chemical, *et al*.) into electrical signals are essential for sensors. Materials for actuators, for reading and writing information, are in the heart of modern technology. Expanding and refining the prediction of polycrystal properties beyond mechanical properties is a task worth undertaking.

Averaging with the orientation distribution function as weighting factor is of common use [6,7]. Classical Voigt [8], Reuss [9] and Hill [10] approximations are standard procedures that researches use as a reference in elasticity investigations [11,12].

Recent investigations have contributed bounds for the predicted properties that are more stringent than those of Voigt and Reuss [13,14]. The search of the effective properties by means of finite elements codes [15], the use of a geometric mean [16,17], self-consistent algorithms [18,19] and full-field theories that take into account the neighboring effects [20] conform the current *state of the art* in the considered research area.

The majority of publications devoted to the calculation of “effective properties” relates to so-called “principal interactions”. Elastic moduli and dielectric constant are “principal” because they link actions (causes) and material responses (effects) associated with the same subsystem (mechanical, electrical) of a given material [21].

Thermal expansion, magnetoelectricity and piezoelectricity, on the other hand, are “coupling” (interactions, properties) because they link actions of one subsystem with responses in another one. Coupling interactions, in the field of polycrystal effective properties, have been scarcely considered [5,22]. To the best of our knowledge, there is no systematic proposal for the prediction of the effective values for coupling properties.

In the present work, with the objective of estimating effective values for polycrystal thermo-elasto-electro-magnetic coupling coefficients, the Voigt, Reuss and Hill approximations for the mentioned interactions are established. As a representative case, piezoelectricity is discussed in some detail. The proposed methodology has been systematized in an extended version of program SAMZ [23].

## 2. Mathematical Background

Consider a single-crystal that is investigated at meso- or macroscopic scale. By “physical property” we understand the magnitude that links an external action with the response of this crystal. In symbols:

**Y** = **K** · **X** (1)

**X** represents the applied action, **Y** is the material response and **K** is the property. In general, **X** and **Y** are tensors with respective ranks *m* and *n*. The property tensor rank is *r = m + n*. As examples of the mentioned regularity we quote the following: *r* = 2: electrical permittivity; *r* = 3: piezoelectricity; *r* = 4: elasticity. Considered tensors may be polar or axial, time-independent or time-reversible. For instance, magnetoelectricity is a well-known case of axial property, linking polar time-independent electric polarization with axial time-reversible magnetic field [24].

A detailed characterization of thermo-elasto-electro-magnetic equilibrium properties, under linear approximations, is given in [25]. The following constitutive equations are established in the mentioned work:

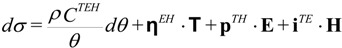
(2)

**S** = **η**^*EH*^*dθ* + **s**^*θEH*^ · **T** + **d**^*θH*^ · **E** + **b**^*θE*^ · **H** (3)

**D** = **P**^*TH*^*dθ* + **d**^*θH*^ · **T** + **ε**^*θTH*^ · **E** + **α**^*θT*^ · **H** (4)

**B** = **i**^*TE*^* dθ* + **d**^*θE*^ · **T** + **α**^*θT*^ · **E** + **μ**^*θTE*^ · **H** (5)

The magnitudes selected as independent variables, or “actions”, are the temperature *θ*, the stress **T** = ║*T_ij_*║ the electric field intensity **E** = ║*E_m_*║ and the magnetic field intensity **H** = ║*H_n_*║. This set of independent variables represent physical actions frequently applied in real-world experiments. The single-crystal nature of the considered material assures that, under homogenous stimuli, the proposed independent variables remain constant in the investigated volume. Dependent variables, or “material responses”, are entropy *σ*, strain **S** = ║*S_ij_*║, electric displacement **D** = ║*D_m_*║ and magnetic induction **B** = ║*B_n_*║. The physical properties are the density *ρ*, the heat capacity *C*, the thermal expansion tensor **η** = ║*η_ij_*║, the pyroelectric and pyromagnetic vectors **p** = ║*p_n_*║ and **i** = ║*i_n_*║, the compliance tensor **s** = ║*s_ijkl_*║, the piezoelectric and piezomagnetic tensors **d** = ║*d_ijm_*║ and **b** = ║*b_ijn_*║, the permittivity **ε** = ║*ε_ij_*║, the permeability **μ** = ║*μ_ij_*║ and the magnetoelectric tensor **α =** ║*α_ij_*║. The supra-indexes in Equations (2)–(5) denote magnitudes considered invariant in the property definition. Following usual conventions, differential symbols corresponding to mechanical and electromagnetic magnitudes are omitted.

Equations (2)–(5) describe four principal interactions (thermodynamic, elastic, electric and magnetic) and twelve coupling interactions, namely: thermal expansion, five piezo-effects (piezocaloric, direct and converse piezoelectric, direct and converse piezomagnetic), four pyro-effects (direct and converse, electric and magnetic) and two magnetoelectric effects (direct and converse).

The configuration of the matrix representing a property tensor is determined by the pertinent point group, as established by the Neumann Principle: *the symmetry of any physical property is at least equal to the structure symmetry* [26].

One intuitive way to describe the properties is by means of the so-called longitudinal surfaces *K*(**h**) [27]. In this graphical characterization, the distance from the origin to the surface represents the longitudinal effect of the action in different directions. We are interested in the expansion of *K*(**h**) in a series of crystal-symmetrized two-dimensional spherical harmonics 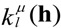 [28]:

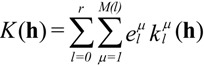
(6)


In Equation (6) the sum over *l* runs up to *l = r*, the rank of the tensor **K**. The sum over *μ* runs up to *M*(*l*), dependent on the crystal point group. The maximum *M*(*l*)* =* 2*l* + 1 corresponds to triclinic crystals. A systematic presentation of the symmetrized spherical harmonics corresponding to all the crystal classes can be found in reference [29]. As illustration, for the tetragonal case exposed below, *M*(*l*) = [*l*/4] + 1. The term [*l*/4] represents the floor function of *l*/4. Piezoelectricity (*l*_max_ = *r* = 3), in a tetragonal crystal, is represented by the sum of two terms, the first one associated with *l* = 1 and the second one with *l* = 3.

The structure and properties of a polycrystal are significantly associated with the orientation distribution of the crystals,* i.e*., the texture. The fundamental statistical descriptor of texture is the orientation distribution function (ODF → *f*(**g**)) [6]. Here we follow Bunge’s formalism [6]:

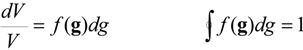
(7)


The orientation of a crystal is described as a point **g** = (*φ_1_, ϕ, φ_2_*) in Euler space. The volume differential in this space is:

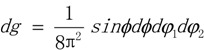
(8)


The ODF is expressed as an expansion in a series of symmetrized tri-dimensional spherical harmonics 
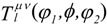
:

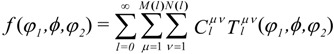
(9)



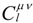
 are expansion coefficients. The limits *M*(*l*) and *N*(*l*) depend respectively on crystal and sample symmetry.

The application of generalized harmonics to the description of textures and to the calculation of average elastic and plastic properties, in different systems, has been reported by [30,31,32].

An important special case in functional (bulk- and nano-) materials is that of axially symmetric, so-called “fiber” textures. Nano-islands, nano-rods (in “parallel” formation) and nano-layers (piled in “series-like” configuration) frequently exhibit the fiber-texture condition. In these cases the ODF role is played by the inverse pole figure (IPF → *R*(**h**)) corresponding to the sample symmetry axis ***z***:

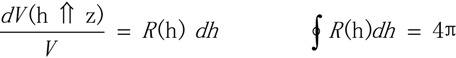
(10)


The IPF is represented by a two-dimensional symmetrized spherical harmonics expansion:

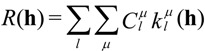
(11)


“Mean” values of action, response and properties of a polycrystal are calculated as follows:

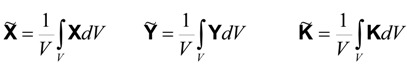
(12)


The weighting factor is the ODF:

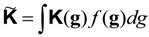
(13)


If the property **K** is described in the longitudinal surface representation, the formalism shown in Equation (13) adopts the following interesting form:

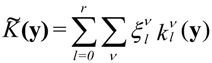
(14)


The 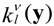 are spherical harmonics adapted to the sample’s symmetry and the coefficients 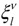 are calculated by:

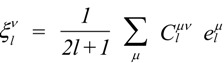
(15)


Index *v* runs up to *N*(*l*). Table 1 shows the dependence of *N*(*l*) with *l* for triclinic, orthorhombic and axial sample symmetries. Triclinic sample symmetry is found mostly in rocks linked with geological studies. Orthorhombic symmetry is characteristic of laminated sheets. Fiber (axial) textures are frequent in functional ceramics.

If the texture shows axial symmetry, Equations (14) and (15) simplify to:

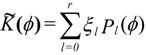
(16)

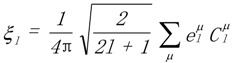
(17)


with 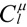 corresponding to Equation (11). *P_l_*(*ϕ*) are the Legendre polynomials.

**Table 1:** Range of summation indexes *N*(*l*) = max *v* in average calculations.

Texture symmetry	Representative samples	*N*(*l*)
Triclinic	Rocks	2*l* + 1
Orthorhombic	Laminated sheets	⌊*l*/2⌋ + 1
Axial	Wires, functional ceramics	1

Equations (15) and (17) represent an important moment in the mathematical analysis of textures. They express, in the symmetrized spherical harmonics terminology, the relationship between single-crystal properties, texture and polycrystal “mean” properties.

*Mean* polycrystal properties represent an approximation to the *effective* magnitudes that are measured in an experiment.

“Global” or “macroscopic” action and response, in a polycrystal case, are 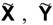, given by Equation (12). By ***effective polycrystal property*** it is understood the magnitude < **K** > that satisfies the following condition:

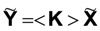
(18)


Mathematically, the following relationship can be proven [6]:

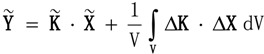
(19)


The *mean* 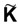 (Equation (13)) represents the *effective* property if the independent variable remains invariant in the sample volume. Effective polycrystal properties not only depend on the distribution of orientations, but also on crystallites’ shapes, sizes and relative positioning,* i.e*., on sample’s stereography.

The influence of sample stereography has been treated extensively for elasticity (a principal interaction). For a polycrystal with a series configuration, the stress can be considered as constant in the sample volume. For this geometry, known as *Reuss* case, it is advisable to apply the constitutive equation **S** = **s**·**T** (**Y** → **S** = strain; **K** → **s** = compliance; **X** → **T** = stress). As ∆**T** = 0, the integral in Equation (15) vanishes and this leads to Equation (14), with = 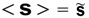.

For parallel configuration, *Voigt* case, the suitable constitutive equation is **T** = **c·S** (**c** = stiffness). Finally, the so-called *Hill approximation* for **s** is:

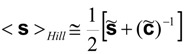
(20)


References [33] and [34] describe representative applications of the VRH approximations to polycrystal elasticity.

## 3. Estimating the Effective Properties for Coupling Interactions. The Piezoelectric Case

To our knowledge, the following systematization has not been divulged previously. Our presentation focuses attention on piezoelectricity, but the fundamental ideas may be applied to other interaction fields in a straightforward manner.

Consider a polycrystalline piezoelectric. Sample texture and single-crystal properties tensors are known. How does one organize a predictive estimation of polycrystal properties?

Moving from single- to polycrystals means losing the structural homogeneity assumed in Equations (2) and (5). Working with polycrystals, if *mean* properties are to be considered as approximations to *effective* properties, requires careful selection of the magnitudes representing actions. Independent variables must remain constant in the whole sample volume. Taking into account the wide diversity of possible polycrystal stereographies, the limiting cases of parallel and series arrangements are worth being considered as reference configurations.

Regarding homogeneity of physical magnitudes, the following considerations apply:

- Thermodynamics: Homogeneity of temperature defines the thermal equilibrium condition for any thermodynamic system.
- Elasticity: In a series configuration, mechanical equilibrium imposes continuity of **T** across inter-crystalline boundaries. In parallel, geometrical integrity leads to continuity of **S**.
- Electricity: In series arrangement, Gauss law applied to boundaries without free charge (**𝛁** · **D** = 0) gives **D** = constant. In parallel, the conservative nature of electrostatic field (**𝛁** × **E** = 0) imposes **E** = constant.
- Magnetism: In series-like polycrystals, Gauss law for magnetism (**𝛁** · **B** = 0) implies **B** = constant. In parallel condition, Ampere law in absence of free currents (**𝛁** × **H** = 0) leads to homogeneity of **H**.

Table 2 summarizes the results of the given analysis:

**Table 2:** Homogeneity conditions for physical magnitudes in polycrystal invariant magnitudes.

Configuration	Thermodynamics	Elasticity	Electricity	Magnetism
Series (Reuss)	Temperature (*θ*)	Stress (**T**)	Electric displacement (**D**)	Magnetic induction (**B**)
Parallel (Voigt)		Strain (**S**)	Field intensity (**E**)	Field intensity (**H**)

Focusing attention into the phenomenon of piezoelectricity, the previous analysis shows that averaging the (most frequently reported) piezoelectric *charge constant* “**d**” would lead to inconsistencies. The same tensor **d** satisfies **S = d**·**E** (Equation (3), converse piezoelectricity) as well as **D** = **d**·**T** (Equation (4), direct piezoelectricity). The first equation would support the consideration of **d** for a Voigt-type (parallel configuration, constant **E**) approximation, while the second would suggest a Reuss-type (series arrangement, constant **T**) averaging.

To avoid inconsistencies like the just mentioned one, the invariance criteria given in Table 2 must be applied. The transformation from the independent variables in Equations (2) and (5) to the required ones is performed by means of Legendre transformations. In the present article the sign conventions of the IEEE standards [35,36] are followed. Equations (21)–(24) present, in expanded matrix notation, the proper constitutive equations for characterizing the principal and coupling properties in a parallel-type (Voigt model) polycrystal.

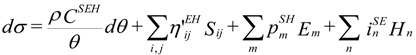
(21)

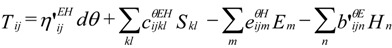
(22)

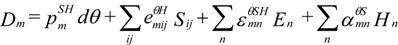
(23)

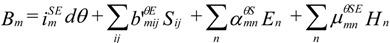
(24)


**Figure 1:**
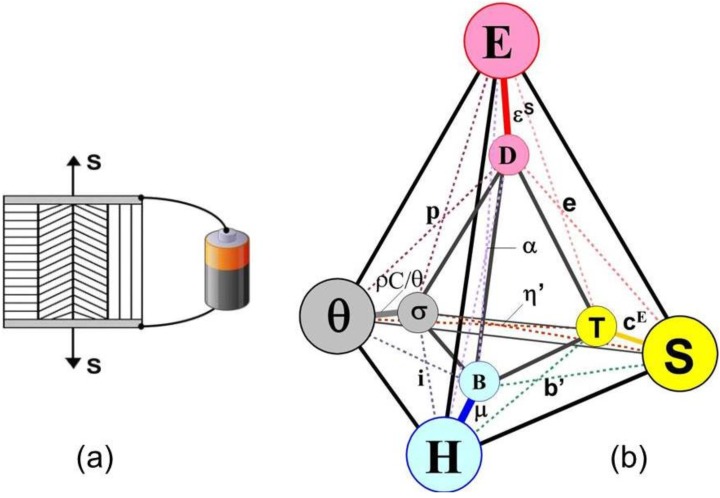
(**a**) A polycrystal in ideal parallel configuration during a (direct or converse) piezoelectric experiment. The actions to be measured are strain and voltage (electric field intensity); (**b**) Graphical representation of principal and coupling interactions. Voigt case.

Figure 1a represents schematically an ideally parallel polycrystal with imposed deformation and applied voltage as external stimuli. Figure 1b shows a graphical representation of the interactions characterized by Equations (21)–(24). The spheres in the outer tetrahedron represent actions (independent variables) while those in the internal tetrahedron describe responses (dependent variables). Links are “principal” and “coupling” properties. The new symbols in Figure 1b denote the following: **c**^E^ = stiffness at constant electric field, **e** = piezoelectric coefficient, **ε**^S^ = permittivity at constant strain,

Equations (25)–(28) represent thermo-elasto-electro-magnetic interactions under the Reuss approximation.

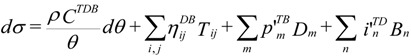
(25)

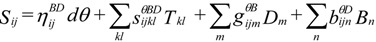
(26)

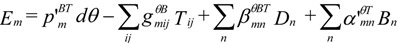
(27)

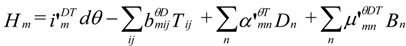
(28)


**Figure 2:**
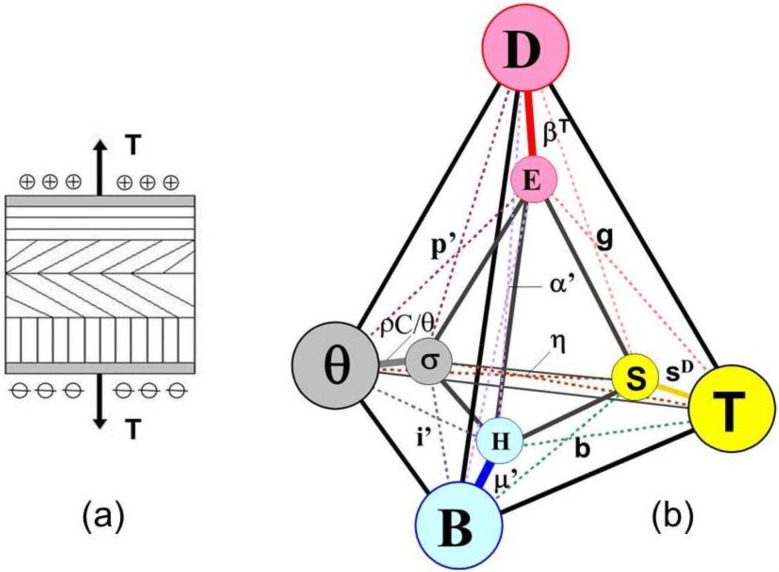
(**a**) A polycrystal in ideal series arrangement during a piezoelectric experiment. Suitable independent variables are stress and electric charge; (**b**) Graphical representation of principal and coupling interactions. Reuss case.

Figure 2a depicts an ideal series polycrystal with external stress and electric charges as appropriate descriptors of external actions. Figure 2b illustrates the interactions expressed in Equations (25)–(28). The new magnitudes are: **β**^T^ = impermittivity at constant stress, **g** = voltage piezoelectric coefficient, **s**^D^ = compliance at constant electric displacement.

Next, we analyze in detail the estimation of polycrystal piezoelectricity. Other coupling properties could be treated by following the same basic ideas. Consider single crystal tensors **s^D^**, **ε^T^** (permittivity at constant stress) and **d** as known magnitudes. The desired goal is to establish the Reuss and Voigt approximations for a textured sample of known ODF.

For the Reuss conditions, the constitutive equations linked with piezoelectricity are:

**S = s^D^ · T + g · D** (29)

**E** = −**g · T** + **β^T^ · D** (30)

Required single-crystal tensors are obtained by application of the equations:

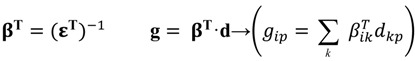
(31)


Tensors** s^D^**, **β^T^** and **g**, or their longitudinal surfaces, are averaged according to the Bunge algorithms, Equations (9), (10) and (12). The magnitudes thereby obtained are denoted 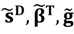.

The Reuss averages for **s**, **ε** and **d** are:

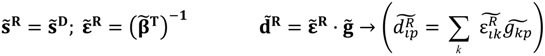
(32)


We turn now to the Voigt case. The piezoelectric equations are:

**T** = **c^E^ · S – e · E** (33)

**D** = **e · S – ε^S^ · E** (34)

Required single-crystal tensors are:

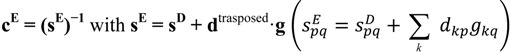
(35)

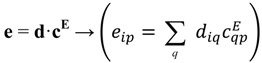
(36)

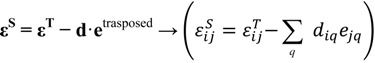
(37)


From single-crystal tensors we obtain poly-crystal ones following Bunge once again. Mean tensors lead to Voigt averages:

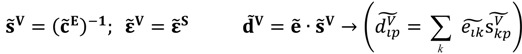
(38)


In case Hill averages are required, they are obtained by averaging the averages. For example:

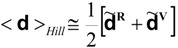
(39)


## 4. SAMZ Program

In order to perform the previous calculations and represent their results in a graphic environment, the authors have developed the application SAMZ, written in MATLAB language. The program computes the properties for piezoelectric samples showing fiber textures. The input data are: (a) a complete set of the elasto-piezo-dielectric tensors associated with the single crystals under study; (b) a model of the crystal structure and the polycrystal texture.

Different sets of single crystal tensor components can be introduced. The program includes routines for calculating, according to the IEEE conversion rules [35], the tensors required for each approximation.

The structure and texture models are specified by the crystal point group, the unit cell dimensions and the inverse pole figure. The input for this last-mentioned data consists of the favored crystal direction and the orientation distribution width. The form of the distribution can be Gaussian or Lorentzian. To establish the multiplicity of the population maxima, the program applies point group symmetry operations. SAMZ displays the IPF graphical representation and characterizes this function by its symmetrized spherical harmonics expansion.

According to the user selection (Voigt or Reuss), the application computes suitable averages for polycrystal properties. By application of exposed mathematical tools, Equations (13)–(17), SAMZ combines single crystal tensors and texture models to estimate the polycrystal properties. Eventually, it also calculates the Hill approximation and represents the corresponding longitudinal surfaces.

## 5. Results and Discussion

### 5.1. A Case Study. Piezoelectricity in PMN-PT

In this section we use the system (1−*x*)·Pb(Mg_1/3_Nb_2/3_)O_3_–*x*PbTiO_3_ (PMN-PT) with composition *x* = 0.3, close to the morphotropic phase boundary (MPB), as an illustration of the method and software described above. The expected goal is a predictive estimate of the piezoelectric coefficients (specially *d_ip_*) for a polycrystal ceramic with a known fiber texture.

When obtaining these ceramics, one looks for fiber textures with maximum population of [0,0,1] crystal direction parallel to the sample symmetry axis [37]. For this purpose, the use of templated grain growth is often employed in the synthesis [38]. The crystals obtained by this method are frequently arranged in a stack of flake-like shapes with the desired texture [39]. The resulting configurations can be represented, with fair approximation, as series alignments. Reuss approximation, therefore, is advisable.

The tensors corresponding to the elasto-piezo-dielectric properties of PMN-PT single crystals, with compositions near to the MPB, have been reported by a number of authors. Published values show some degree of dispersion. This is due to composition, symmetry and stress differences among the samples, as well as to variations in the applied polarizing fields.

In this work, single-crystal **ε^T^** (=**ε**_0_**K**^T^), **d** and **s^D^** tensors reported by [40] will be used as reference. **K**^T^ is the dielectric constant. The above-mentioned authors report the tensor properties corresponding to *x* = 0.30. A pseudo-tetragonal symmetry, point group *C*_4v_ = 4 mm, is assumed.

Considered matrices are presented in Equations (40)–(42).

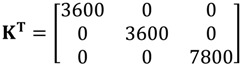
(40)

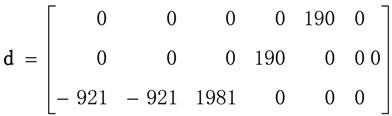
(41)

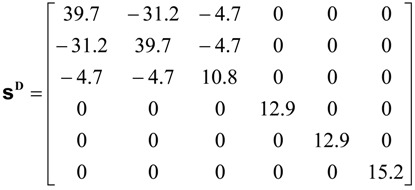
(42)


**Figure 3:**
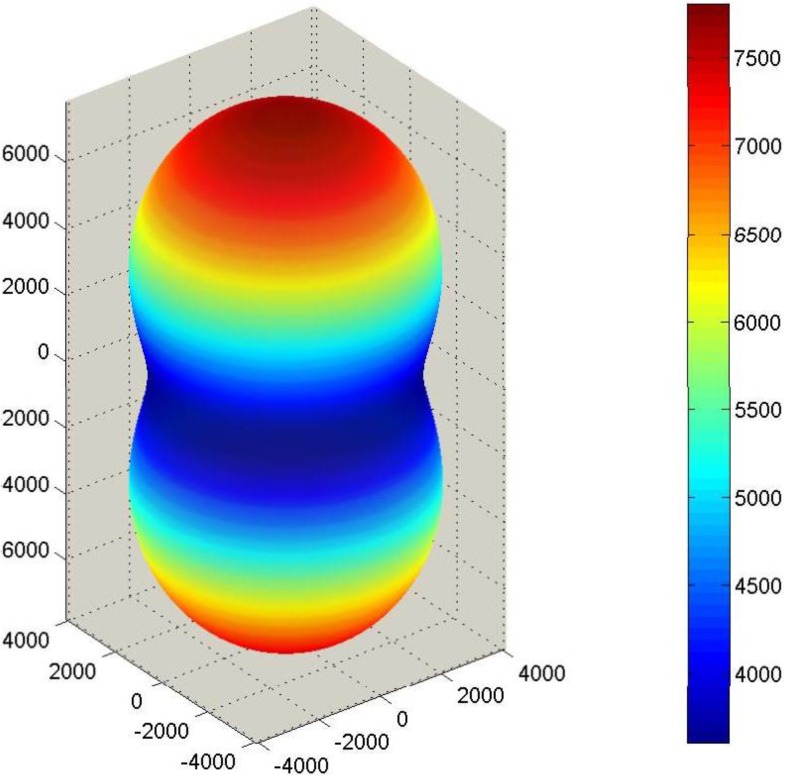
Longitudinal surface representation, *K*^T^(**h**), for the dielectric constant of a PMN-PT single crystal.

**Figure 4:**
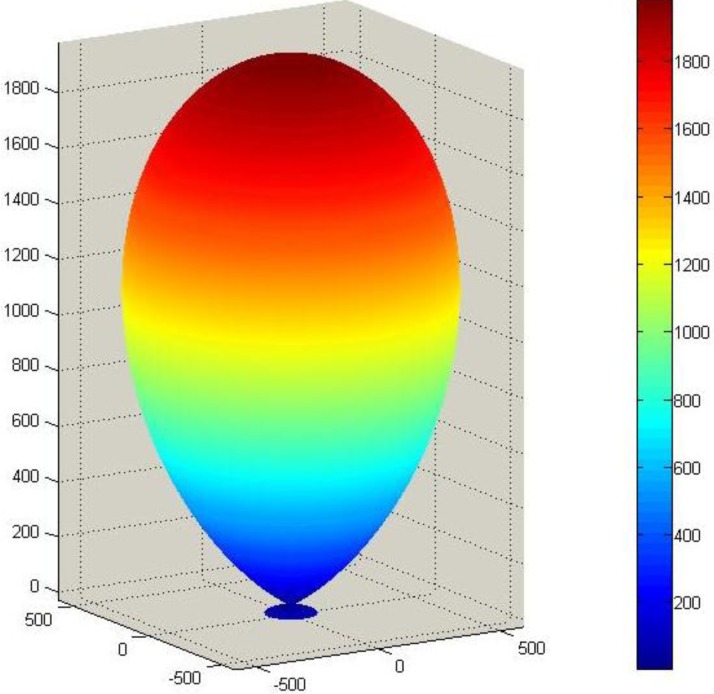
Longitudinal surface representation, *d*(**h**) (10^−12^ C/N), for the piezoelectric charge constant of a PMN-PT single-crystal.

**Figure 5:**
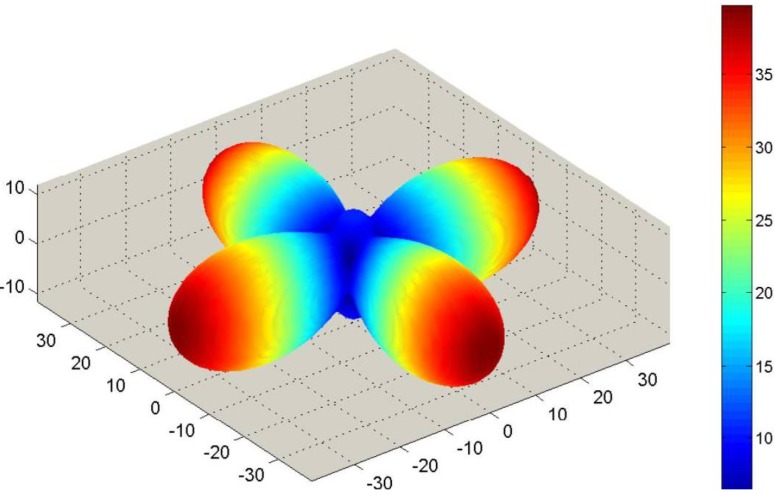
Longitudinal surface representation, *s***^D^**(**h**) (10^−12^ m^2^/N), for the compliance of PMN-PT.

Corresponding SAMZ surface representations are shown in Figure 3, Figure 4 and Figure 5.

**Figure 6:**
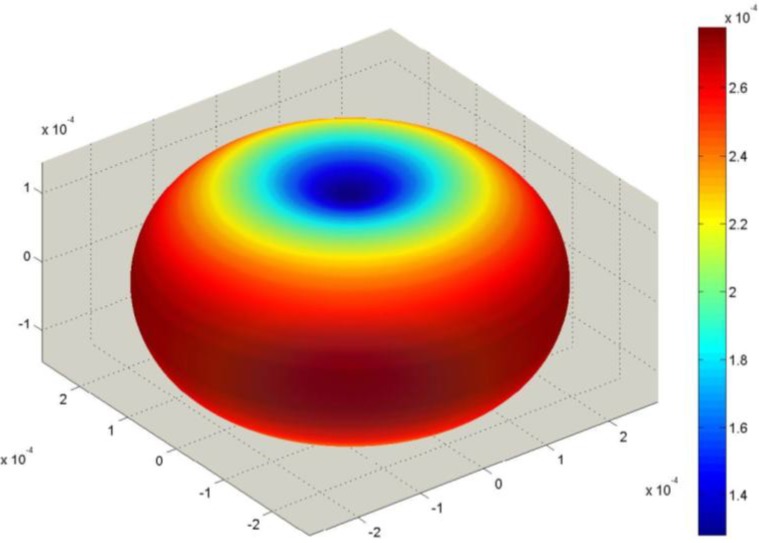
Longitudinal surface representation, *ε_0_***β**^T^ (**h**), for the impermittivity of a PMN-PT single crystal.

**Figure 7:**
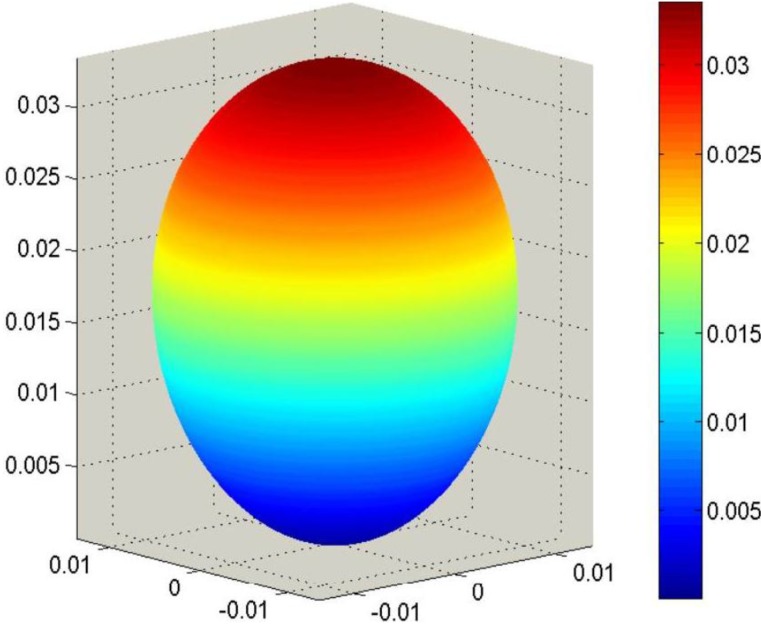
Longitudinal surface representation, *g*(**h**) (Vm/N), for the voltage piezoelectric constant of a PMN-PT single crystal.

To average under the Reuss approximation, the tensors **s**^D^, **β**^T^ and **g** are required. Using Equation (19), the initial data can be properly converted. The single-crystal surfaces for *ε_0_***β**^T^ and **g** are represented in Figure 6 and Figure 7.

Several authors [39,41,42] have reported comparable degrees of texture for PMN-PT in the MPB. Describing texture through the so-called “Lotgering factor”, which estimates the fraction of textured material, is a common practice. Representative published Lotgering factors are in the interval *f* ≈ 70%–90%. According to [43], we rather convert this Lotgering-like description to a formal characterization of the orientation distribution. We represent fiber textures by the sample symmetry axis IPF. Equation (43) describes the IPF of a PMN-PT hypothetical sample with a Gaussian component in [0,0,1], with distribution width Ω = 25°.

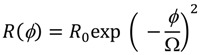
(43)


The proposed IPF, applied as correction factor to a randomly oriented powder x-ray diffraction pattern, leads to a pattern of diffraction intensities comparable to the ones in the mentioned articles.

The combination of single-crystal properties (Figure 3, Figure 6 and Figure 7) and texture (Equation (43)) leads to Reuss averages. Looking particularly for the effective value of **d**, Reuss averages of **g** and 
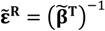
 are required (see Equation (32)). The necessary calculations (Equations (6), (11), (14) and (15)) are performed by SAMZ.

**Figure 8:**
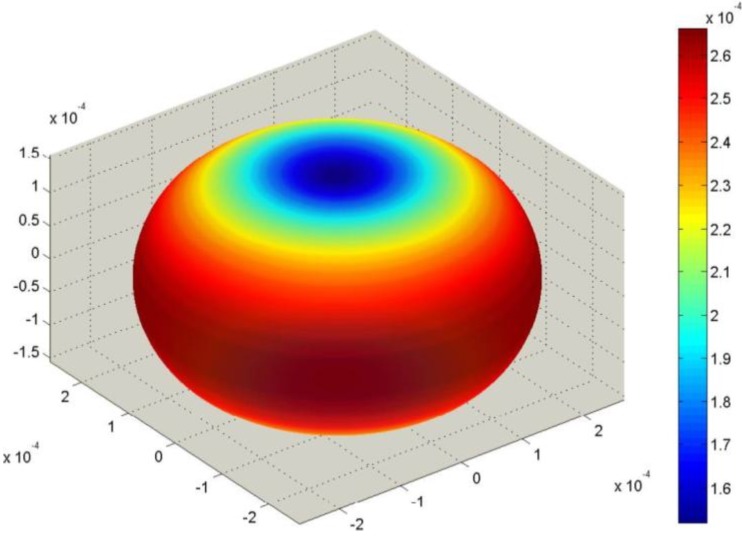
Longitudinal surface representation, *ε_0_β*^T^(**h**), for the impermittivity of a PMN-PT textured polycrystal.

**Figure 9:**
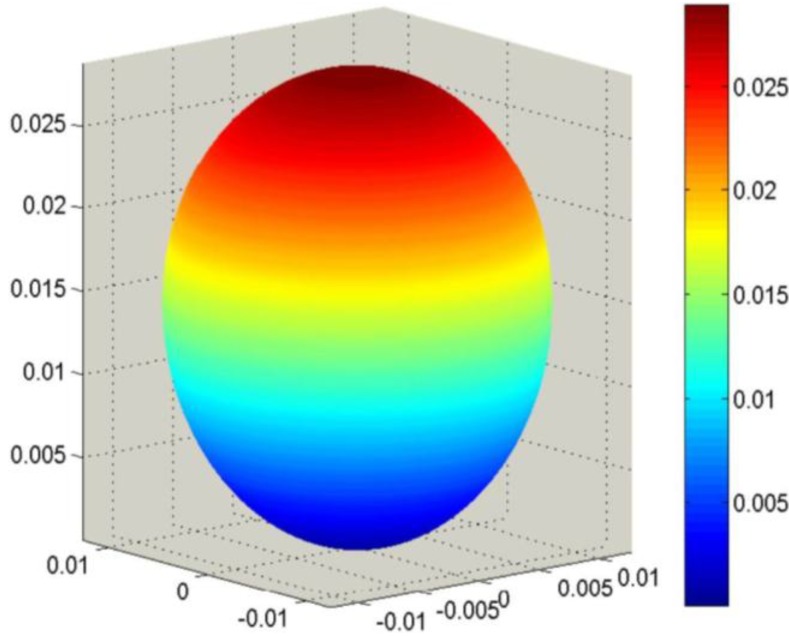
Longitudinal surface representation, *g*(**h**), for the voltage piezoelectric constant of a PMN-PT textured polycrystal.

Figure 8 and Figure 9 show the calculated polycrystal surfaces for impermittivity 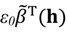 and voltage piezoelectric constant 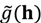.

Part of the information included in Figure 8 and Figure 9 are the following values:

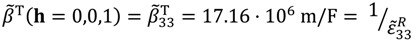
(44)

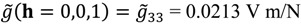
(45)


Calculated properties allow us to deliver an estimate of the frequently looked-for polycrystal 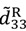. In our particular case the calculation is rather simple:

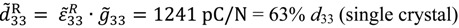
(46)


Table 3 presents some *d*_33_ values recently reported for PMN-PT samples that show similar composition and texture as those of our model.

**Table 3:** Observed piezoelectric charge coefficients *d*_33_. PMN-PT at the morphotropic phase boundary (MPB).

Reference	*f*	*d*_33_ (pC/N)
[41]	0.90	1150
[39]	0.70	1600
[42]	0.82	870

### 5.2. Discussion

Texture, described statistically by the ODF (IPF), is one of the factors affecting the physical properties. Its influence is important, but not unique. Additional aspects that should be taken into account when predicting properties are:

To what extent model single-crystal tensors correspond to the actual investigated material. The PMN-PT family illustrates the fact that small changes in composition or physicochemical conditions involve significant variations in single-crystal properties.

Several factors such as the porosity of the sample or the internal stresses can change the polycrystal properties, even if the intrinsic characteristics of each crystallite remain unaffected.

The stereography (morphology, size of crystals, series-parallel arrangement and interfaces) can show a wide diversity for the same ODF. Stereography changes also involve changes in the macroscopic properties.

As often happens in science, to improve a reasonable result into a refined one, it requires a major effort. (In X-ray crystallography practice, this is an everyday affair. To lower intensities uncertainties from 10% to 1% takes 100 times longer measuring times.) For the problems discussed in this article, to slightly exceed the predictions of Voigt, Reuss and Hill, the measurement and calculation effort is considerable. Finite-elements procedures and self-consistent methods do mean progress, but are significantly expensive in workload.

Unambiguous “self-consistent” solutions, dependent only on the ODF, can backfire. In the real world different samples, with different effective properties, can have the same ODF. In these cases the aim should be to seek the best representation of the stereography and to average accordingly.

For coupling properties, even VRH are not yet systematized. In this paper, we propose a novel methodology, based on traditional VRH, to estimate acceptable approximations for effective coupling properties. The suggested procedure has been coded into an accessible computer program.

## 6. Conclusions

Crystallographic texture significantly impacts the effective values of the polycrystalline properties. Knowledge of single-crystal properties and of the ODF allows a predictive estimation of the above-mentioned properties.

The spherical harmonics expansion of texture descriptors and crystal properties allows a systematization of the necessary calculations. This systematization has resulted in the program SAMZ, accessible by Internet.

The consideration of the sample stereography should complement and guide the calculation of averaged properties. Each one, among traditional VRH approaches, has its fitting case.

Factors beyond the texture should be taken into account for a correct prediction.

## References

[1] Yan Z., Zaman M., Jiang L. (2011). Thermo-electro-mechanical analysis of a curved functionally graded piezoelectric actuator with sandwich structure. Materials.

[2] Friak M., Counts W.A., Ma D., Sander B., Holec D., Raabe D., Neugebaure J. (2012). *Theory-*guided materials design of multi-phase Ti-Nb alloyswith bone-matching elastic properties. Materials.

[3] Bunge H.J., Kiewei R., Reinert Th., Fritsche L. (2000). Elastic properties of polycrystals—Influence of texture and stereology. J. Mech. Phys. Solids.

[4] Böhlke T., Jöchen K., Kraft O., Löhe D., Schulze V. (2010). Elastic properties of polycrystalline microcomponents. Mech. Mater..

[5] Sheng G., Bhattacharyya S., Zhang H., Chang K., Shang S.L., Mathaudhu S.N., Liu Z.K., Chen L.Q. (2012). Effective elastic properties of polycrystals based on phase-field description. Mater. Sci. Eng. A.

[6] Bunge H.J., Morris P.R. (1982). Texture Analysis in Materials Science: Mathematical Methods.

[7] Gruber J.A., Brown S.A., Lucadamo G.A. (2011). Generalized Kearns texture factors and orientation texture measurement. J. Nucl. Mater..

[8] Voigt W. (1910). Lehrbuch der Kristallphysik:(Mit Ausschluss der Kristalloptik) (in German).

[9] Reuss A. (1929). Berechnung der flieβgrenze von mischkristallen auf grund der plastizitätsbedingung für einkristalle (in German). Z. Angew. Math. Mech..

[10] Hill R. (1952). The elastic behaviour of a crystalline aggregate. Proc. Phys. Soc. Sect. A.

[11] Kocks U.F., Tomé C.N., Wenk H.-R. (2000). Texture and Anisotropy: Preferred Orientations in Polycrystals and Their Effect on Materials Properties.

[12] Topolov V.Y., Bowen C.R. (2009). Effective electromechanical properties in piezo-composites. Electromechanical Properties in Composite Based on Ferroelectrics.

[13] Knezevic M., Kalidindi S.R. (2007). Fast computation of first-order elastic–plastic closures for polycrystalline cubic-orthorhombic microstructures. Comput. Mater. Sci..

[14] Fast T., Knezevic M., Kalidindi S.R. (2008). Application of microstructure sensitive design to structural components produced from hexagonal polycrystalline metals. Comput. Mater. Sci..

[15] Gawad J., Van Bael A., Eyckens P., Samaey G., Van Houtte P., Roose D. (2013). Hierarchical multi-scale modeling of texture induced plastic anisotropy in sheet forming. Comput. Mater. Sci..

[16] Matthies S. (2010). On the combination of self-consistent and geometric mean elements for the calculation of the elastic properties of textured multi-phase samples. Solid State Phenom..

[17] Chateigner D., Ricote J., Pardo L., Ricote J. (2011). Quantitative texture analysis of polycrystalline ferroelectrics. Multifunctional Polycrystalline Ferroelectric Materials.

[18] Ramírez M., Nava-Gómez G.G., Sabina F.J., Camacho-Montes H., Guinovart-Díaz R., Rodríguez-Ramos R., Bravo-Castillero J. (2012). Enhancement of Young’s moduli and auxetic windows in laminates with isotropic constituents. Int. J. Eng. Sci..

[19] Camacho-Montes H., Sabina F.J., Bravo-Castillero J., Guinovart-Díaz R., Rodríguez-Ramos R. (2009). Magnetoelectric coupling and cross-property connections in a square array of a binary composite. Inter. J. Eng. Sci..

[20] Lebensohn R.A., Kanjarla A.K., Eisenlohr P. (2012). An elasto-viscoplastic formulation based on fast Fourier transforms for the prediction of micromechanical fields in polycrystalline materials. Int. J. Plast..

[21] Du Q., Li J., Nothwang W., Cole M.W. (2006). The dielectric behavior of polycrystalline ferroelectric films with fiber textures. Acta Mater..

[22] Muñoz-Romero A., Aquino De Los Ríos G., Domínguez-Barrera P., Fuentes-Montero L., Camarillo-Cisneros J., Camacho-Montes H., Fuentes-Montero M.E., Montero-Cabrera M.E., García-Guaderrama M., Fuentes-Cobas L. (2011). From nano to bulk: Computer- and synchrotron-aided investigation of the structure-properties relationship. Integr. Ferroelectr..

[23] Muñoz-Romero A., de los Ríos G.A., Fuentes-Cobas L. SAMZ Home Page. http://crystal.cimav.edu.mx/samz/.

[24] Eerenstein W., Mathur N., Scott J.F. (2006). Multiferroic and magnetoelectric materials. Nature.

[25] Fuentes-Cobas L., Matutes-Aquino J., Fuentes-Montero M., Buschow K.H.J. (2011). Magnetoelectricity. Handbook of Magnetic Materials.

[26] Fuentes-Cobas L., Fuentes-Montero M. (2008). La Relación Estructura-Simetría-Propiedades en Cristales y Policristales (in Spanish).

[27] Nye J. (1985). The Physical Properties of Crystals.

[28] Raymond O., Fuentes L., Gómez J.I. (1996). Surface representation of polycrystal physical properties: All crystal classes, simple average approximation. Textures Microstruct..

[29] Gómez J. (1996). Computer-oriented real spherical harmonics for texture and properties analyses. Texture Stress Microstruct..

[30] Knezevic M., Kalidindi S.R., Mishra R.K. (2008). Delineation of first-order closures for plastic properties requiring explicit consideration of strain hardening and crystallographic texture evolution. Int. J. Plast..

[31] Wu X., Proust G., Knezevic M., Kalidindi S.R. (2007). Elastic–plastic property closures for hexagonal close-packed polycrystalline metals using first-order bounding theories. Acta Mater..

[32] Shaffer J.B., Knezevic M., Kalidindi S.R. (2010). Building texture evolution networks for deformation processing of polycrystalline fcc metals using spectral approaches: Applications to process design for targeted performance. Int. J. Plast..

[33] Graczykowski B., Mielcarek S., Breczewski T., No M., San-Juan J., Mroz B. (2013). Martensitic phase transition in Cu-14%Al-4% Ni shape memory alloys studied by Brillouin light scattering. Smart Mater. Struct..

[34] Berryman J.G. (2013). Computing elastic constants for random polycrystals of orthotropic MgSiO_3_, related polymorphs, and CaIrO_3_ analogs. J. Comput. Phys..

[35] Meitzler A., Tiersten H., Warner A., Berlincourt D., Couqin G., Welsh F. (1987). IEEE Standard on Piezoelectricity “ANSI/IEEE Std 176–1987”.

[36] Ehrlich S., Ballato A., Butler J., Clark A., Moffett M., Pozzo W., Ricketts D., Tims A. (1990). IEEE Standard on Magnetostrictive Materals: Piezomagnetic Nomenclature “IEEE Std 319–1990”.

[37] Slodczyk A., Colomban P. (2010). Probing the nanodomain origin and phase transitionmechanisms in (un)poled PMN-PT single crystals and textured ceramics. Materials.

[38] Andreeta E.R.M., dos Santos H.F.L., Andreeta M.R.B., Lente M.H., Garcia D., Hernandes A.C., Eiras J.A. (2007). Anisotropy on SrTiO_3_ templated textured PMN–PT monolithic ceramics. J. Eur. Ceram. Soc..

[39] Kwon S., Sabolsky E.M., Messing G.L., Trolier-McKinstry S. (2005). High strain, <001> textured 0.675Pb(Mg_1/3_Nb_2/3_)O_3_–0.325PbTiO_3_ ceramics: Templated grain growth and piezoelectric properties. J. Am. Ceram. Soc..

[40] Zhang R., Jiang W., Jiang B., Cao W. (2002). Elastic, dielectric and piezoelctric coefficients of domain engineered 0.70Pb(Mg_1/3_Nb_2/3_)O_3_–0.30PbTiO_3_ single crystal. AIP Conf. Proc..

[41] Sabolsky E.M., Trolier-McKinstry S., Messing G.L. (2003). Dielectric and piezoelectric properties of <001> fiber-textured 0.675Pb (Mg_1/3_Nb_2/3_)O_3_-0.325PbTiO_3_ ceramics. J. Appl. Phys..

[42] Zhao W., E L., Ya J., Liu Z., Zhou H. (2012). Synthesis of high-aspect-ratio BaTiO_3_ platelets by topochemical conversion and fabrication of textured Pb(Mg_1/3_Nb_2/3_)O_3_-32.5PbTiO_3_ ceramics. Bull. Korean Chem. Soc..

[43] Brosnan K.H., Messing G.L., Meyer R.J., Vaudin M.D. (2006). Texture measurements in <001> fiber-oriented PMN–PT. J. Am. Ceram. Soc..

